# Complete remission of central nervous system manifestations of IgG4-related disease with rituximab – a case report

**DOI:** 10.1177/17562864231186091

**Published:** 2023-07-18

**Authors:** Aleksi J. Sihvonen, Sini M. Laakso, Olli Tynninen, Heikki Saaren-Seppälä, Mervi Löfberg

**Affiliations:** Department of Neurology, Neurocenter, Helsinki University Central Hospital, Haartmaninkatu 4, P.O. Box 340, 00029 Helsinki, Finland; Clinical Neurosciences, Neurology, University of Helsinki and Brain Center, Helsinki University Hospital, Helsinki, Finland; Cognitive Brain Research Unit, Department of Psychology and Logopedics, Faculty of Medicine, University of Helsinki, Finland; Centre of Excellence in Music, Mind, Body and Brain, University of Helsinki, Finland; Clinical Neurosciences, Neurology, University of Helsinki and Brain Center, Helsinki University Hospital, Helsinki, Finland; Department of Pathology, Helsinki University Hospital and University of Helsinki, Helsinki, Finland; Department of Ophthalmology, Helsinki University Hospital and University of Helsinki, Helsinki, Finland; Clinical Neurosciences, Neurology, University of Helsinki and Brain Center, Helsinki University Hospital, Helsinki, Finland

**Keywords:** brain, case report, IgG4-related disease, pachymeningitis, rituximab, parenchymal

## Abstract

IgG4-related disease (IgG4-RD) is an emerging immune-mediated chronic fibrotic disease characterized by tumour-like mass formation. Reports of brain parenchymal involvement in IgG4-RD are rare and complete treatment-related remission of lesions has never been reported. Here, we present a woman in her mid-50s who developed headache and seizures. Brain magnetic resonance imaging revealed frontal bilateral pachymeningitis and a left frontal lobe parenchymal lesion, and pathologic findings were consistent with an IgG4-RD central nervous system manifestation. She had a history of tumour-like growth around the right optic nerve, orbital and maxillary cavities treated successfully with corticosteroids 28 years ago, and was receiving infliximab as a maintenance therapy for uveitis for the last 14 years. After initial high-dose corticosteroid treatment, the patient was treated with rituximab, and after 3 months, the patient presented with complete remission of IgG4-RD lesions and associated symptoms. This case illustrates the chronic, decades-spanning nature of IgG4-RD, and a complete response to rituximab even with intracerebral mass lesions that had emerged despite the use of infliximab, a therapy previously reported successful in IgG4-RD.

## Introduction

IgG4-related disease (IgG4-RD) was defined only in 2003 as a clinical entity and is regarded an immune-mediated fibrotic disease with a progressive and chronic nature.^1^ The disease is characterized by mass-forming lesions consisting of lymphoplasmacytic infiltrates rich in IgG4-positive plasma cells and sclerosis, and in most patients, high-serum IgG4 levels.^2^ The most recognized presentations of IgG4-RD are type 1 autoimmune pancreatitis and salivary gland disease, but multiple organs are affected in 60−90% of all patients.^3^ Published cases of IgG4-RD have reported involvement of almost all organs, including descriptions of central nervous involvement in form of pachymeningitis,^4^ orbital pseudotumor,^5^ inflammatory enlargement of pituitary gland,^5,6^ pterygopalatine fossa,^6^ and cranial nerve enlargement.^5^ However, previous studies reporting involvement of brain parenchyma in IgG4-RD are scarce.

In 2014, Regev and colleagues reported a case of IgG4-RD with multiorgan involvement including several cortical and subcortical brain lesions.^7^ The patient presented with spastic hemiparesis and dementia responding to high-dose corticosteroid treatment followed by rituximab. The treatment prevented the patient from having new lesions, but complete radiological or neurological recovery was not reported. More recently, De Maria and colleagues reported a case presenting with seizures and cerebral masses related to IgG4-RD.^8^ The patient showed favorable response to glucocorticoid treatment, but radiological or long-term outcomes were not reported.

## Case report

Here, we report the case of a woman in her mid-50s who presented with progressive headache and 3 months later with focal seizures that arrested speech production for the duration of 1 to 2 min. She was admitted to Helsinki University Hospital neurology ward in January 2022. Twenty-eight years earlier, the patient had presented with tumour-like growth around the right optic nerve, orbital, and maxillary cavities. Two separate biopsies via craniotomy revealed fibrotic inflammatory proliferation and the patient was diagnosed with Tolosa-Hunt syndrome. Corticosteroids were administered with a good response. Fifteen years later, regrowth of the tumour was observed on imaging, but biopsy could confirm only fibrosis, and the specific diagnosis remained unclear. The patient responded well to treatment with corticosteroids and the observed tumour-like growth disappeared. Four years after the initial diagnosis of Tolosa-Hunt syndrome, the patient developed chronic bilateral iritis and uveitis. Due to chronic uveitis, the patient had been treated in Helsinki University Hospital Department of Ophthalmology with glucocorticoids, but cystoid macular edema (CME) persisted and steroid-sparing medication (methotrexate and azathioprine) were tried. Slowly, the situation subsided with intravitreous corticosteroid injections and regular infliximab-infusions for 14 years preceding admission to neurology ward.

**Figure 1.**
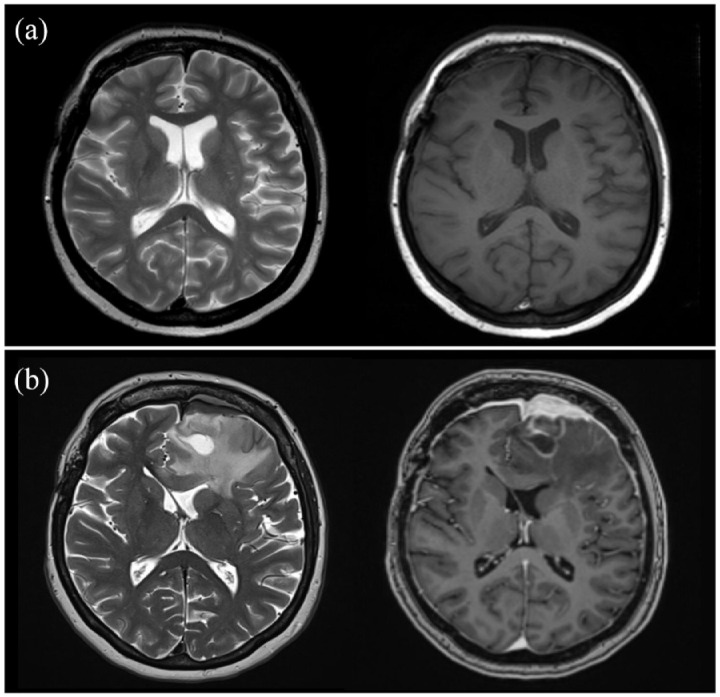
Magnetic resonance imaging findings (a) 14 years earlier and (b) upon admittance.

Upon admittance, no significant neurological findings were observed in the clinical evaluation, but during monitoring, the patient presented with a focal seizure that arrested her speech production for 1 to 2 min. Paraphasias, dysarthria, or accompanying sensorimotor symptoms were not observed. A brain magnetic resonance imaging (MRI) scan with MRI angiography was acquired that revealed frontal bilateral pachymeningitis and left frontal lobe parenchymal inflammatory infiltrate (Figure 1). The neuroradiological findings were considered to be primarily associated with IgG4-RD or secondarily with sarcoidosis or granulomatosis. Levetiracetam was initiated.

An electroencephalogram showed widespread intermittent theta and delta wave abnormalities in the left hemisphere. While clear epileptiform activity was not observed, the clinical presentation was in concordance with symptomatic focal epilepsy due to the parenchymal findings. She was discharged and referred to the department of neurosurgery for a diagnostic biopsy and to neurological outpatient clinic for further care.

Thereafter seizures reoccurred, and the patient was again admitted to the neurological ward. MRI scan showed that the previously observed pachymeningitis and left frontal lobe parenchymal inflammatory infiltrate had progressed and were now accompanied with oedema. Lacosamide was added to antiepileptic medication. A preponed biopsy was performed in the left frontal lobe lesion and the adjacent meninges followed by initiation of methylprednisolone treatment.

**Figure 2.**
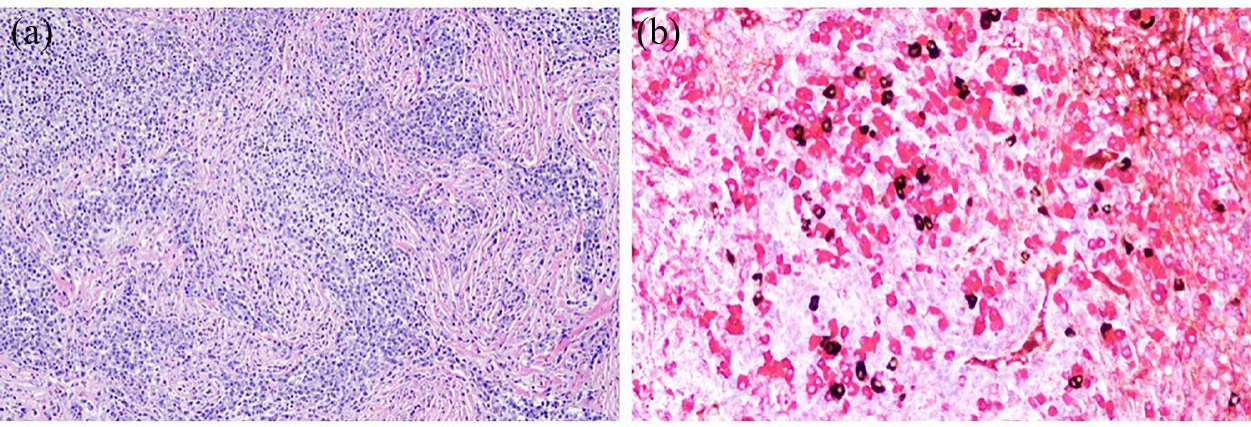
Histologic findings of the biopsy. (a) Dural biopsy shows heavy lymphoplasmacytic inflammation and fibrosis (hematoxylin-eosin staining, original magnification 200×). (b) Immunohistochemical double staining shows increased number of IgG4 + plasma cells (brown) with abundant IgG + plasma cells (red) (original magnification 400x).

The biopsy revealed thickened and fibrotic dura with dense lymphoplasmacytic inflammation (Figure 2). Perivascular lymphocytes were present but obliterative phlebitis was not seen. Cortical brain tissue showed macrophages and reactive gliosis with perivascular lymphocytes and plasma cells. Granulomas or necrosis were not present in dura or in brain parenchyma. The inflammatory cells consisted mostly of CD20+ B-lymphocytes, CD3+ T-lymphocytes, and CD138+ plasma cells. Further immunohistochemical workup did not show evidence of mucosa associated lymphoid tissue lymphoma. Infectious agents were not detected by microbial staining. Immunohistochemical IgG4 staining showed up to 40 IgG4+ plasma cells per high-power field. IgG4+/IgG+ plasma cell ratio was 10%. The biopsy fulfilled 2 of the 3 histological features of IgG4-RD consensus criteria (lymphoplasmacytic infiltrate and fibrosis).^9^ The number of IgG4-positive plasma cells was increased (>10 cells per high-power field); however, IgG4 to IgG plasma cell ratio was <40%.

**Figure 3.**
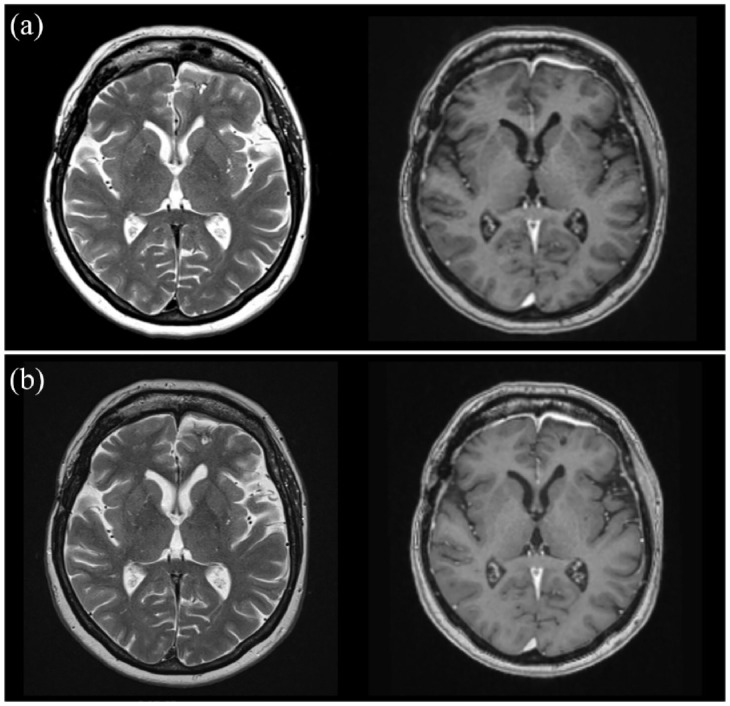
Magnetic resonance imaging findings (a) 3 months and (b) 7 months after initiation of the treatment.

To treat both the chronic uveitis and the observed central nervous system infiltrates, infliximab was replaced with rituximab, which was administered in 1000 mg given twice with a 2-week interval. During the follow-up, the patient remained seizure-free and the headache had resolved. After 3 months of rituximab-treatment, the previously observed brain parenchymal changes and pachymeningitis were no longer observable in the brain MRI with gadolinium enhancement nor had new lesions emerged (Figure 3). Extensive neuropsychological examination of the patient was stated normal. To rule out other affected organs, a full-body computed tomography (CT) scan was acquired that revealed no IgG4-RD findings or other abnormalities. After 7 months from initiation of rituximab, there were no neurological symptoms or findings, and follow-up brain MRI was normal (Figure 3). Antiepileptic medication was reduced back to levetiracetam alone. Rituximab is administered 500 mg every 6 months as a maintenance therapy. Patient’s vision has remained stable, but after changing infliximab to rituximab, CME has started to reoccur and intravitreous injections might be required in the future.

## Discussion

Central nervous system involvement in IgG4-RD is rare. The prevalence of meningeal involvement is approximately 2% of overall clinical manifestations in IgG4-RD,^4,10^ and true central nervous system involvement (i.e. pachymeningoencephalitis) even more so.^7,8,11^ The disease often responds well to corticosteroids, also when there are meningeal manifestations.^12^ However, in severe, refractory, and steroid-dependent cases inclusion of rituximab in the treatment regime has shown promise.^7,13^ In parenchymal infiltrates, such reports are scarce.^7,8,11^ To our knowledge, this is the first report of complete clinical and radiological remission of probable IgG4-RD pachymeningoencephalitis after rituximab treatment. Infliximab has also been reported beneficial in treating IgG4-RD^14^ but our patient had long-lasting infliximab-therapy with confirmed high blood concentration and no autoantibody production against the medication at admittance, thus suggesting suboptimal effect against preventing manifestations of the central nervous system.

Biopsy is considered the gold standard for establishing a diagnosis in meningeal or brain parenchymal IgG4-RD.^9^ Characteristic histopathological findings include fibrosis, lymphoplasmacytic infiltration with IgG4-positive plasma cells and obliterative phlebitis. For meninges, a threshold of 10 IgG4-positive plasma cells per high-power field has been recognized as diagnostic,^7,10^ whereas higher thresholds might apply different organs, for example, 100 IgG4-positive plasma cells per high-power field in skin.

Our case report highlights the chronic nature of IgG4-RD, with almost 30 years from the first likely manifestation to setting the diagnosis based on modern histopathological analysis. Initially, the patient was diagnosed with Tolosa-Hunt syndrome, which is a rare neuro-ophthalmic manifestation characterized by granulomatous inflammation. As neuro-ophthalmological manifestations are quite commonly observed in IgG4-RD,^15^ supported also by our patient, and IgG4-RD is almost indistinguishable from Tolosa-Hunt syndrome in its ocular presentation,^16^ it remains unknown whether the case was IgG4-RD from the beginning. Infliximab-therapy was unable to prevent the central nervous system manifestations, although was successful in treating uveitis. Our findings show complete and maintained remission with rituximab in IgG4-RD affecting the brain parenchyma, supporting the role of CD20+ B cells as disease drivers. A recent finding of a highly clonal CD8 + T cell infiltration within an IgG4-RD brain parenchymal lesion suggests a close interplay of B and T cells, and thus the benefit of rituximab might be linked to decreased antigen presentation by memory B cells.^17^ Further prospective treatment trials with rituximab are warranted.

## Declarations

**Ethics approval and consent to participate:** A written informed consent was obtained from the patient. Ethics approval is not applicable.

**Consent for publication:** A written informed consent was obtained from the patient, covering the publication of medical patient data and images.

**Availability of data and materials:** Not applicable.
